# Premarital sexual practice and associated factors among adolescents in the refugee camps in Tigray, northern Ethiopia

**DOI:** 10.1186/s13104-019-4459-x

**Published:** 2019-07-15

**Authors:** Hailay Gebreyesus, Tesfay Berhe, Zemichael Welegebriel, Tewolde Wubayehu, Gebretsadik Hailemariam, Gebretsadkan Gebrekirstos, Mebrahtu Teweldemedhin

**Affiliations:** 1 Department of Public Health, College of Health Science, Aksum University, P.O. Box 298, Aksum, Ethiopia; 2 School of Medicine, College of Health Science, Aksum University, Aksum, Ethiopia; 3 Department of Medical Laboratory Sciences, College of Health Science, Aksum University, Aksum, Ethiopia; 4 Administration for Refugee and Returnee Affairs Adiharsh Eritrean Refugee Camps Health and Neutrino Coordinator, Adiharsh, Addis Ababa, Ethiopia

**Keywords:** Premarital sexual practice, Health risks, Refugee adolescent, Tigray, Ethiopia

## Abstract

**Objective:**

Premarital sexual practice during adolescence time may lead to different sexual and reproductive health problems including HIV/AIDS. This study was aimed to assess the prevalence of premarital sexual practice and associated factors among adolescents living in the refugee camps in Tigray, northern Ethiopia.

**Result:**

The prevalence of premarital sexual practice was 47.6% [95% CI (43.3%, 51.9%)]. The age (mean ± SD) of the study participants was 17.4 ± 2.71 (15–24 years) and 68.8% of them were males. Being in the age group 14–19 years [AOR = 4.50, 95% CI (2.29–8.84)] or 20–24 years [AOR = 9.76, CI (4.58–20.82)], living with friends [AOR = 5.24, 95% CI (2.28–12.05)] or alone [AOR = 5.72, 95% CI (2.69–12.19)], being in primary school [AOR = 6.93, 95% CI (3.08–15.57)] or college [AOR = 4.33, CI (1.44–13.02)], getting pocket money [AOR = 4.14, 95% CI (2.31–7.41)], parents being either divorced [AOR = 5.11, 95% CI (2.42–10.80)] or widowed [AOR = 3.52, 95% CI (1.69–7.33)], alcohol consumption [AOR = 1.99, 95% CI (1.20–3.38)] were independent predictors of premarital sexual practice among the adolescents.

**Electronic supplementary material:**

The online version of this article (10.1186/s13104-019-4459-x) contains supplementary material, which is available to authorized users.

## Introduction

According to the World Health Organization, an adolescent is an individual aged from 10 to 19 years. Adolescent is the time of transition from childhood to adulthood and characterized by significant physical, mental, and behavioral changes that may place their life at high risk [1, 2]. Refugees are people including adolescents who have been forced to leave their homes to avoid the effects of armed conflict, generalized violence, violations of human rights, natural or human-made disasters and who have crossed an international border; they are highly vulnerable to different risky sexual behaviors [3].

Premarital sexual activities among adolescents are increasing from time to time worldwide particularly in developing countries like Ethiopia [4]. The premarital sexual practice among those adolescents particularly living in refugee camps is unprotected and predisposes for different sexual and reproductive health problems [5]. For instance, about 45% of all new HIV infections globally occurred among adolescents aged 15–24 years. In Africa, 60% of all new HIV infections occur in adolescents aged 15–19 years [6–8]. Each year, about 15 million adolescents aged 15–19 years gave birth; a large proportion of these pregnancies were unwanted, as many as 4 million experienced unsafe abortion, and up to 100 million were affected by STDs [9–11].

Studies in Ethiopia showed that 45% of the total birth occurs among adolescents aged 15–19 years of which 24.4% of them had an unwanted pregnancy and 89% with a history of abortion [12, 13]. The WHO report showed that sexual violence is frequently occurring among adolescents in developing countries [14]. About 54% in Ethiopia and 48% in Bangladesh adolescents aged 15–19 years had experienced sexual violence within the last 12 months respectively [14]. Another study conducted in Kenya showed that 40% sexually experienced adolescent girls and 65% of boys were having more than one sexual partner and only a few of them were using condoms [15].

Different studies conducted in developing countries revealed that substance use, peer influence, living arrangement, age, having pocket money, watching pornographic video and misconceptions on sexual risk behaviors were factors associated with premarital sexual practice and its complications [5, 16–19]. Although there were very limited studies, the risks of sexual and reproductive health problems are higher among adolescents living in the refugee camps than for adolescents in the general community [20]. Thus, the aim of this study was to assess the prevalence of premarital sexual practice and its associated factors among Adolescents living in refugee camps, in Tigray, northern Ethiopia.

## Main text

### Methods

#### Study setting and period

A community-based cross-sectional study was conducted in the refugee camps, located in North Tigray region, northern Ethiopia, at 1023 km from Addis Ababa. There are four refugee camps in Shire operation namely, Adi Harush, Shimelba, Mai-Aini, and Hitsats. According to the United Nation Higher Commissioner for Refugee (UNHCR) on 31 January 2018, there are a total of 44,683 refugees in the four identified camps of whom 13,800 are adolescents aged from 15–24 years. Each refugee camp has one health center and one elementary school. The participants of this study were unmarried adolescents 10–24 years old of the selected zones of the refugee camps, who had a refugee identification card. Those adolescents aged 10–24 years who were unable to communicate due to their physical and mental illnesses were excluded from the study. The study was done from April to May 2018.

#### Sample size and sampling technique

The sample size was calculated using a single population proportion formula considering the prevalence of premarital sexual practice among high school adolescents 21.1% [21], a margin of error 5% and adding 5% for non-response rate. The final sample size was 536 with a design effect of 2. Stratified sampling technique was used to include participants from the four refugee camps. After the proportional allocation to each refugee camp, simple random sampling was used to select individuals using the respective camp registry as a sampling frame.

#### Data collection tools and procedure

An anonymous, structured questionnaire was developed after reviewing relevant literatures that reviewed key variables as well as earlier studies on premarital sexual practice among adolescents [4, 7, 13, 16, 20–22]. The questionnaire and consent document was first developed in English and then translated into the local language (Tigrigna) and finally retranslated into English by another translator to check the consistency. The questionnaire had different parts which included socio-demographic, premarital sexual practice, risky behaviors, and related variables. Training was given for the data collectors and supervisors on how to conduct the interview and do the supervision.

Five percent of the questionnaire was pre-tested among adolescents in the nearby community for clarity and ascertain internal consistency. After analyzing the pre-test result, necessary modifications were made accordingly before using the questionnaire in the actual survey. Data were collected by four clinical nurses under the supervision of two public health experts.

The primary outcome variable for the study was measured by the answers to the question: “Have you ever had sexual intercourse?” The adolescents were free to respond either “Yes” or “No”. Those who responded “Yes” and were never married were regarded as having premarital sexual practice.

#### Data processing and analysis procedures

The coded data were entered into Epi-info Version 3.5.3 and then exported to SPSS Version 21 for analysis. Descriptive statistics were used to describe the continuous and categorical variables. Crude association between dependent and independent variables was assessed by bivariable logistic regression. Multivariable logistic regression analysis was carried out to control confounding variables. In the multivariable logistic regression analysis, a *p* value < 0.05 and the 95% CI were considered to determine the statistical significance; adjusted odds ratio (AOR) was used to determine the strength of association.

### Results

#### Socio-demographic characteristics of the participants

A total of 536 adolescents participated in this study with a response rate of 100%. Majority of them, 369 (68.8%) were males. The age (mean ± SD) of the study participants was 17.4 ± 2.71 (15–24 years). Based on their living arrangements, 250 (46.6%) of the adolescents were living either alone or with friends (Table 1).

**Table 1:** Socio-demographic characteristics of the study participants in Tigray, refugee camps, northern Ethiopia, 2018 (N = 536)

Variables	Frequency	Percent (%)
Sex		
Male	369	68.8
Female	167	31.2
Age		
< 14	158	29.5
14–19	234	43.7
20–24	144	26.9
Living with arrangement		
With father and mother	131	24.4
Father only or mother only	155	28.9
Relatives/friends	109	20.3
Alone	141	26.3
Family marital status		
They live together	215	40.1
Separated	118	22.0
Divorced	94	17.5
Widowed	109	20.3
Previous residence		
Urban	199	37.1
Rural	337	62.9
Religion		
Orthodox	309	57.6
Muslim	116	21.6
Catholic	90	16.8
Protestant	21	3.9
Educational status		
Unable to read and write	119	22.2
Only read and write	158	29.5
Primary school (grade 1–8)	150	28.0
Secondary school (grade 9–12)	80	14.9
College and above	29	5.4
Ethnicity		
Tigrigna speakers	495	92.4
Other^a^	41	7.6
Pocket money		
Yes	144	26.9
No	392	73.1

^a^Kunama, Amhara, Somali and Afar

#### Experiences of premarital sexual practices

The prevalence of premarital sexual practice was 47.6% [95% CI (43.3%, 51.9%)]. Majority of the adolescents, 347 (64.7%) had girl/boyfriends. Most of the adolescents (53.7%) who have had premarital sex were in the age group of 14 to 19 years. One hundred sixty-seven (65.5%) of them had sex with just acquaintance to their sexual partner. The reason for their first sex was the desire to practice sex in 103 (40.4%) and peer pressure in 88 (34.5%) of the respondents. No contraceptive method was used by 101 (39.6%) of the adolescents who have had sex (Table 2).

**Table 2:** Distribution of experiences of adolescents on premarital sexual practices, in Tigray, refugee camps, northern Ethiopia May, 2018

Variables	Frequency	Percent (%)
Have had ever sex (n = 536)		
No	281	52.4
Yes	255	47.6
Had boy/girl friend (n = 536)		
Yes	347	64.7
No	189	35.3
Age of first sex (n = 255)		
< 14	92	36.1
14–19	137	53.7
20–24	26	10.2
Relationship to first sexual partner (n = 255)		
Acquaintances	167	65.5
Friend	88	34.5
Reason for first sex (n = 255)		
Fall in love	34	13.3
Desire to practice sex	103	40.4
To get money and other gifts	30	11.8
Peer pressure	88	34.5
Motivation for premarital sex (n = 255)		
Watching pornography	79	31.0
Watching erotic films	93	36.5
Being intoxicated/drunk	67	26.3
I don’t know	16	6.3
Did you use any contraceptive methods during your last sex (n = 255)		
Yes	154	60.4
No	101	39.6
Reason for not using contraceptives		
Trust partner	44	26.0
Ashamed to ask partners	6	3.6
Ashamed to buy	64	37.9
Partner objected	9	5.3
No sexual experience	46	27.2

#### Risk behaviors of premarital sex

Regarding substance use, about 226 (42.2%) of the respondents had the experience of smoking any tobacco products. About 105 (19.6%) of them had the experience of chewing chat. Regarding alcohol consumption, 339 (63.2%) had the experience of drinking any type of alcohol and from those about 127 (48.7%) of them said that they had sexual practice after drinking alcohol and only 29 (22.8%) of them used condom during sexual practice after drinking alcohol (Additional file 1: Table S1).

#### Source of information and discussion about sexuality and reproductive health

From the 536 study participants, 276 (51.5%) of them responded that they have had information about sexuality and reproductive health issues and their major sources of information were health professionals 100 (36%) followed by family/friends 78 (28.3%) (Additional file 2: Table S2).

#### Factors associated with premarital sexual practice

Results of the multiple logistic regression model showed that age of 14–19 years [AOR = 4.50, 95% CI (2.29–8.84)] and 20–24 years [AOR = 9.76, CI (4.58–20.82)], primary education [AOR = 6.93, 95% CI (3.08–15.57)], having monthly pocket money [AOR = 4.14, 95% CI (2.31–7.41)], living arrangement {living with their relatives/friends [AOR = 5.24, 95% CI (2.28–12.05)] and living alone [AOR = 5.72, 95% CI (2.69–12.19)]}, alcohol consumption [AOR = 1.99, 95% CI (1.20–3.38)], and family marital status {divorced [AOR = 5.11, 95% CI (2.42–10.80)] or widowed [AOR = 3.52, 95% CI (1.69–7.33)]}.were found to be significantly associated with premarital sexual practice (Table 3).

**Table 3:** Multivariate logistic regression on premarital sexual practice among adolescents in Tigray, refugee camps, northern Ethiopia, May, 2018

Variables	Pre-marital sex		COR (95%, CI)	AOR (95%, CI)
	No	Yes		
	n (%)	n (%)		
Sex				
Male	218 (59.1)	151 (40.9)	1	1
Female	63 (37.7)	104 (62.3)	2.38 (1.64–3.47)	1.83 (0.94–3.56)
Age				
<14	96 (60.8)	62 (39.2)	1	1
14–19	121 (51.7)	113 (48.3)	1.45 (.96–2.18)	*4.50 (2.29*–*8.84)*
20–24	64 (44.4)	80 (55.6)	1.94 (1.22–3.06)	*9.76 (4.58*–*20.82)*
Living condition				
With father and mother	97 (74.0)	34 (26.0)	1	1
Father or mother only	91 (58.7)	64 (41.3)	2.00 (1.21–3.32)	1.46 (0.78–2.71)
Relatives/friends	41 (37.6)	68 (62.4)	4.73 (2.73–8.20)	*5.24 (2.28*–*12.05)*
Alone	52 (36.9)	89 (63.1)	4.88 (2.91–8.21)	*5.72 (2.69*–*12.19)*
Education status				
Unable to read and write	75 (63.0)	44 (37.0)	1	1
Only read and write	102 (64.6)	56 (35.4)	0.94 (0.57–1.54)	0.62 (0.32–1.20)
Primary school	51 (34.0)	99 (66.0)	3.31 (2.00–5.47)	*6.93 (3.08*–*15.57)*
Secondary school	40 (50.0)	40 (50.0)	1.71 (0.96–3.03)	*2.17 (1.00*–*4.69)*
College and above	13 (44.8)	16 (55.2)	2.10 (.92–4.77)	*4.33 (1.44*–*13.02)*
Family marital status				
They live together	145 (67.4)	70 (32.6)	1	1
Separated	80 (67.8)	38 (32.2)	0.98 (0.61–1.59)	0.46 (0.21–1.01)
Divorced	20 (21.3)	74 (78.7)	7.66 (4.33–13.56)	*5.11 (2.42*–*10.80)*
Widowed	36 (33.0)	73 (67.0)	4.20 (2.57–6.86)	*3.52 (1.69*–*7.33)*
Pocket money				
No	234 (59.7)	158 (40.3)	1	1
Yes	47 (32.6)	97 (67.4)	3.06 (2.04–4.57)	*4.14 (2.31*–*7.41)*
Ever consumption alcohol				
No	134 (68.0)	63 (32.0)	1	1
Yes	147 (43.4)	192 (56.6)	*2.78 (1.92*–*4.02)*	*1.99 (1.20*–*3.38)*

Odds ratio and CI written in italics indicates statistical significance at p value < 0.05

### Discussion

This community-based cross-sectional study tried to assess the premarital sexual practice and its associated factors among adolescents who lived in the refugee camps in Tigray, northern Ethiopia. In this study, about half of the adolescents (47.6%) in the refugee camps have had premarital sexual practice. Even though there are limited studies about premarital sexual practice particularly among refugee adolescents at the national and local level, the finding of this study is higher than the previous study conducted in Ethiopia among high school adolescent which was 33% [22]. The higher prevalence among refugee adolescents might be due to their difficult living conditions; they did not have permanent residence and they might not be benefited from the sustainable health educations programs about sexual reproductive problems. On the other hand, the finding is similar with another survey conducted in Ethiopia among youth which was almost 50% [23]. This result may imply that there is high probability of youths in refugee who can easily engage in sexual practice.

Age of respondents was associated with the premarital sexual practice; those adolescents whose age was between 14 and 19 years were 4.5 times more likely to start premarital sex compared to adolescents < 14 years of age [AOR = 4.50, 95% CI (2.29–8.84)] and those adolescents whose age was between 20 and 24 years were 9.76 times more likely to start premarital sex compared to adolescents < 14 years of age [AOR = 9.76, CI (4.58–20.82)]. This finding is in agreement with previous studies conducted in Ethiopia among adolescents [24, 25].

Living arrangement of the respondent was significantly associated with premarital sexual practice. Those adolescents who were living with their relatives/friends were 5.24 times more likely to start premarital sex compared to adolescents living with their mother and father [AOR = 5.24, 95% CI (2.28–12.05)] and those adolescents who were living alone were 5.72 times more likely to start premarital sex compared to adolescents living with their mother and father [AOR = 5.72, 95% CI (2.69–12.19)]. Previous researches had indicated that parental involvement affects adolescent behavior primarily through monitoring. Parents, who spend more time supervising their children have children who engage in fewer risky and premarital sexual behaviors [26, 27].

Educational status of the respondents was also associated with premarital sexual practice; those adolescents who attended primary school were 6.93 times more likely to start premarital sex compared to adolescents who were unable to read and write [AOR = 6.93, 95% CI (3.08–15.57)] and being college and above holder was 4.33 times more likely to start premarital sex compared to adolescents who were unable to read and write [AOR = 4.33, CI (1.44–13.02)]. The current finding is not consistent with previous studies conducted elsewhere [12, 21, 28].

This study had also identified the likelihood of premarital sexual practice was higher among adolescents whose parents were either divorced or widowed. Moreover, respondents having pocket money were 4.14 times more likely to start premarital sex compared to adolescents who did not have pocket money [AOR = 4.14, 95% CI (2.31–7.41)]. Other studies identified that a number of social factors exerted a large measure of influence on the attitude of refuge adolescents towards sexual practice [28, 29]. Especially adolescents living in the refugee camp with poor monitoring around the refuge environment and refugee peers provide adolescents the opportunity to expose them to unhealthy behavior contributing to poor decision-making including substance use [30]. Regarding substance use, respondents who consumed alcohol were 1.99 times more likely to engage on premarital sexual practice than non-users [AOR = 1.99, 95% CI (1.20–3.38)] which is in agreement with other studies [28, 31, 32]. In the general, the prevalence of premarital sexual practice was high in the study group. Age, living with friends/alone, being in primary/secondary schools or college and above, getting pocket money, having divorced/widowed parents and alcohol consumption were predictors of premarital sexual practice. Considering the high prevalence of early sexual intention, there should be sustainable community-based health education to reduce the premarital sexual practice among refugee adolescents.

## Limitation of the study

As it was a cross sectional study, causality may not be established and owing to the sensitive nature of the outcome variable, the current reported prevalence may under estimate the real prevalence on the ground and also this study was conducted within few refugee camps in Tigray regional state on limited numbers of adolescents who were living at the selected refugee camps which means the findings may not be generalizable to the overall Ethiopian adolescents living at the selected camps or other refugee camps.

## Additional files

**Additional file 1: Table S1.** Distribution of adolescents by risk behaviors in Tigray, refugee camps, northern Ethiopia, May, 2018.

**Additional file 2: Table S2.** Distribution of adolescents by source of information about sexuality and reproductive health issues, in Tigray, refugee camps, northern Ethiopia, May, 2018.

## Data Availability

The datasets on which conclusion was made is available in the form of Microsoft Excel. It is available on reasonable request.

## Abbreviations

AIDS: acquired immunodeficiency syndrome
DHS: Demographic and Health Survey
HIV: human immunodeficiency virus
NGO: Non-Governmental Organization
RH: reproductive health
STD: sexually transmitted disease
STIs: sexually transmitted infections
USA: United State of America
WHO: World Health Organization
