# Supplementary material for: Premarital sexual practice and associated factors among adolescents in the refugee camps in Tigray, northern Ethiopia
Source: BMC Res Notes. 2019 Jul 15;12:415. doi: 10.1186/s13104-019-4459-x (PMC6631621; doi:10.1186/s13104-019-4459-x)
Supplement: Supplementary file 1 — Additional file 1: Table S1. Distribution of adolescents by risk behaviors in Tigray, refugee camps, northern Ethiopia, May, 2018. [file 13104_2019_4459_MOESM1_ESM.docx]

| Variables | Frequency(n=536) | Percent [%] |
| --- | --- | --- |
| Ever smoke any tobacco products | | |
| Yes | 226 | 42.2 |
| No | 310 | 57.8 |
| Have ever chew chat | | |
| Yes | 105 | 19.6 |
| No | 431 | 80.4 |
| Drink any alcohol products | | |
| Yes | 339 | 63.2 |
| No | 197 | 36.8 |
| Had sexual practice after drinking alcohol(n=261) | | |
| Yes | 127 | 48.7 |
| No | 134 | 51.3 |
| Use condom during sex after drinking alcohol(n=127) | | |
| Yes | 29 | 22.8 |
| No | 98 | 77.2 |
