# Supplementary material for: Premarital sexual practice and associated factors among adolescents in the refugee camps in Tigray, northern Ethiopia
Source: BMC Res Notes. 2019 Jul 15;12:415. doi: 10.1186/s13104-019-4459-x (PMC6631621; doi:10.1186/s13104-019-4459-x)
Supplement: Supplementary file 2 — Additional file 2: Table S2. Distribution of adolescents by source of information about sexuality and reproductive health issues, in Tigray, refugee camps, northern Ethiopia, May, 2018. [file 13104_2019_4459_MOESM2_ESM.docx]

| Variables | Frequency(n=536) | Percent [%] |
| --- | --- | --- |
| Have had information about sexuality and reproductive health | | |
| Yes | 276 | 51.5 |
| No | 260 | 48.5 |
| Sources of information(n=276) | | |
| Family/relatives/friends | 78 | 28.3 |
| Teachers, health professionals | 100 | 36.0 |
| From mass-medias | 60 | 21.7 |
| Others | 38 | 14.0 |
| Discussion about sexual and reproductive health with Family | | |
| Yes | 234 | 43.7 |
| No | 302 | 56.3 |
| Discussion sexual issues with friends | | |
| Yes | 213 | 39.7 |
| No | 323 | 60.3 |

Others: Social Medias
